# Differentiation, Transcriptomic Profiling, and Calcium Imaging of Human Hypothalamic Neurons

**DOI:** 10.1002/cpz1.786

**Published:** 2023-06-01

**Authors:** Hsiao-Jou Cortina Chen, Simone Mazzaferro, Tian Tian, Iman Mali, Florian T. Merkle

**Affiliations:** 1 Metabolic Research Laboratories, Wellcome Trust MRC Institute of Metabolic Science, University of Cambridge, Addenbrooke’s Hospital, Cambridge, UK; 2 Wellcome Trust–Medical Research Council Cambridge Stem Cell Institute, University of Cambridge, Cambridge, UK

**Keywords:** differentiation, human, hypothalamus, neuron, obesity, pluripotent stem cell, POMC, protocol

## Abstract

Neurons in the hypothalamus orchestrate homeostatic physiological processes and behaviors essential for life. Human pluripotent stem cells (hPSCs) can be differentiated into many types of hypothalamic neurons, progenitors, and glia. This updated unit includes published studies and protocols with new advances in the differentiation, maturation, and interrogation by transcriptomic profiling and calcium imaging of human hypothalamic cell populations. Specifically, new methods to freeze and thaw hypothalamic progenitors after they have been patterned and before substantial neurogenesis has occurred are provided that will facilitate experimental flexibility and planning. Also included are updated recipes and protocols for neuronal maturation, with details on the equipment and methods for examining their transcriptomic response and cell-autonomous properties in culture in the presence of synaptic blockers. Together, these protocols facilitate the adoption and use of this model system for fundamental biological discovery and therapeutic translation to human diseases such as obesity, diabetes, sleep disorders, infertility, and chronic stress.

**Basic Protocol 1:** hPSC maintenance

**Basic Protocol 2:** Hypothalamic neuron differentiation

**Support Protocol 1:** Cortical neuron (control) differentiation

**Basic Protocol 3:** Neuronal maturation

**Support Protocol 2:** Cryopreservation and thawing of neuronal progenitors

**Support Protocol 3:** Quality control: Confirmation of hypothalamic patterning and neurogenesis

**Support Protocol 4:** Bulk RNA sequencing of hypothalamic cultures

**Basic Protocol 4:** Calcium imaging of hypothalamic neurons using Fura-2AM

**Alternate Protocol:** Calcium imaging of green fluorescent hypothalamic neurons using Rhod-3 AM

## Introduction

The hypothalamus is a highly conserved brain region responsible for a wide array of homeostatic processes (Swaab et al., 1993). Distinct hypothalamic functions are controlled by unique neuronal cell types, often via neuropeptides that have potent effects on behavior and physiology. For example, neurons producing agouti-related peptide (AGRP) or pro-opiomelanocortin (POMC) stimulate or inhibit feeding behavior, respectively (Cone, 2006; Fan et al., 1997; Ollmann et al., 1997; Zhan et al., 2013). Neurons that produce corticotropin-releasing hormone (CRH) sit at the apex of the hypothalamic-pituitary-adrenal (HPA) axis that regulates stress responses (Aguilera & Liu, 2012; Zoumakis & Chrousos, 2010), and neurons that produce thyrotropin-releasing hormone (TRH) regulate energy expenditure by affecting the activity of the thyroid gland (Hollenberg, 2008; Lechan & Fekete, 2006). Melanin-concentrating hormone (MCH) is a neuropeptide produced by hypothalamic neurons that regulate sleep and metabolism (Ferreira et al., 2017; Konadhode et al., 2013; Qu et al., 1996), and neurons producing hypocretin/orexin (HCRT) are essential for normal sleep regulation and are lost in the sleep disorder narcolepsy (Nixon et al., 2015; Peyron et al., 2000; Thannickal et al., 2000). There is a pressing need to better understand how the abnormal function of hypothalamic neurons contributes to human disease. For example, obesity, thought to be largely a disease of the brain, affects nearly half of adults in Western countries and significantly decreases lifespan (Locke et al., 2015; Ng et al., 2014).

To enable the study of live human hypothalamic neurons, protocols have been developed (Merkle et al., 2015; Wang et al., 2015) to differentiate human pluripotent stem cells (hPSCs), including human embryonic stem cells (hESCs) and human induced pluripotent stem cells (hiPSCs), into hypothalamic neurons. These differentiation protocols were designed by applying understanding of the signaling pathways known to pattern the developing ventral forebrain (Puelles & Rubenstein, 2003; Sussel et al., 1999) and by building upon previously established neuronal directed differentiation protocols (Chambers et al., 2009; Maroof et al., 2013; Shi et al., 2012).

Human stem-cell-derived hypothalamic neurons recapitulate many of the essential properties of their counterparts *in vivo* (Merkle et al., 2015; Wang et al., 2015, 2017; Yamada-Goto et al., 2017), making them an excellent scientific model for a number of reasons. First, hPSCs and their derivatives have diploid genomes broadly representative of those found in human populations (Adewumi et al., 2007; Amps et al., 2011; Thomson et al., 1998), an essential consideration when seeking to model the effect of human genetic variants on cellular phenotypes. The ability to readily modify the human genome using CRISPR/Cas9 and other gene-editing tools facilitates the generation of reporter cell lines to study cell types of interest and to generate isogenic disease models (Hendriks et al., 2016; Hockemeyer & Jaenisch, 2016; Merkle & Eggan, 2013; Ran et al., 2013). Second, in contrast to the complex environment encountered in the brain, the reduced complexity of a cell culture model system enables the effects of compounds on neuronal function to be directly tested. The accessibility of cells *in vitro* also facilitates time-lapse imaging studies as well as physiological and optogenetic studies. Finally, hPSCs provide a theoretically limitless supply of hypothalamic neurons, which can be differentiated on a large scale. The generation of large cell numbers enables proteomic studies and facilitates the development of functional screens based on neuronal activity.

Here, we provide an updated version of our previously published protocols (Chen et al., 2021; Kirwan et al., 2017; Merkle et al., 2015), which utilize a chemically defined medium for greater reproducibility and improved neuronal maturation, and also detail methods for transcriptomic profiling and calcium imaging. We hope that these protocols will enable more groups to study the basic biology of hypothalamic neurons and develop new disease models to facilitate the development of treatments for a range of human diseases.

***BASIC PROTOCOL 1***

## hPSC Maintenance

hPSCs are prone to spontaneous differentiation and genetic instability if not handled appropriately (Ronen & Benvenisty, 2012) and should be maintained in conditions that stably promote pluripotency. The presence of spontaneously differentiated cells in hPSC cultures is likely to interfere with efficient hypothalamic differentiation, and the presence of unwanted mutations will complicate downstream analyses. Detailed methods for hPSC culture are provided elsewhere (International Stem Cell Initiative Consortium et al., 2010; Ludwig & Thomson, 2007; Santos et al., 2016).

**Figure 1:**
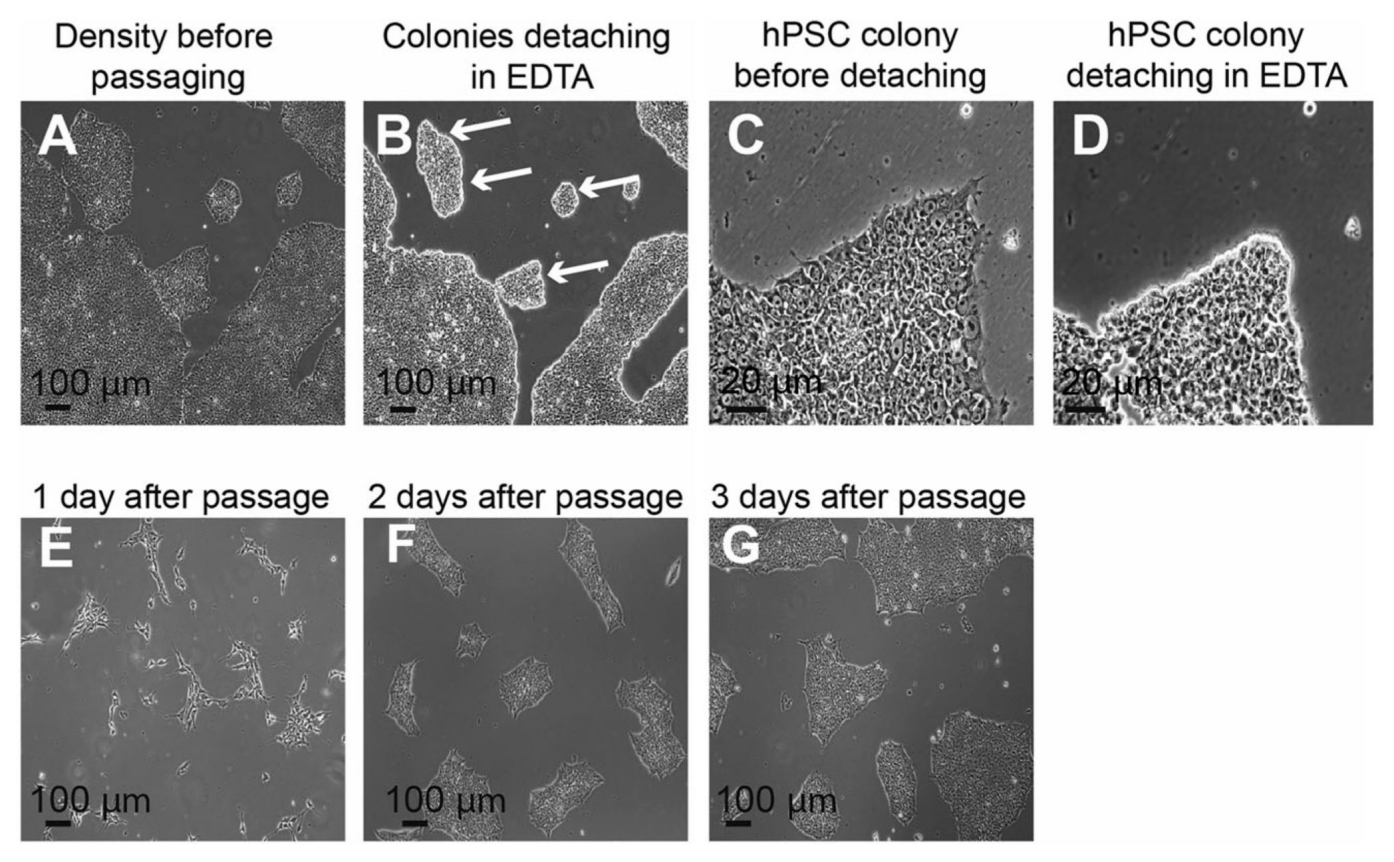
hPSC culture and maintenance. (**A**) hPSCs at 80% confluence before passaging. (**B**) After brief incubation in EDTA, the edges of the colonies begin to detach, taking on a phase-bright appearance (arrows). (**C**) 40× magnification image of an hPSC colony before passaging. (**D**) After incubation in EDTA, borders between individual hPSCs should become clear, phase bright, and well separated. (**E**) hPSCs on the day after a passage, showing a typical density after passaging. Note that hPSCs extend more processes in the presence of the ROCK inhibitor Y-27632, giving colonies a jagged appearance. (**F**) Colonies acquire smoother borders upon withdrawal of Y-27632 the next day. (**G**) Expansion of hPSC colonies is evident 3 days after passaging. Colonies of undifferentiated hPSCs should have cells that are uniform in morphology.

hPSC maintenance is summarized in Figure 1. Briefly, hPSCs are maintained in the absence of antibiotics under feeder-free conditions, monitored and fed daily with complete medium changes, and gently passaged once they reach 70%-80% confluence. Cell lines should be routinely monitored for pathogens such as mycoplasma, as well as for the acquisition in culture of unwanted mutations (Amps et al., 2011; Baker et al., 2016; Merkle et al., 2017). Also note that work with hESCs is restricted in some countries and institutions, but they can be replaced with hiPSCs for most applications. Indeed, selecting a commonly used reference hiPSC line such as KOLF2.1J can facilitate reproducibility and data sharing between groups (Pantazis et al., 2022). Below, we describe conditions for hPSC maintenance with StemFlex, but other feeder-free media and supplements can be used as well. If your group prefers other media, we suggest that cells be adapted for at least 3 passages in StemFlex on a substrate of Geltrex or Matrigel before undergoing neuronal differentiation using the protocols described here.

### Materials

Geltrex LDEV-Free Reduced Growth Factor Basement Membrane Matrix (Thermo Fisher Scientific, cat. no. A1413202)
DMEM/F12 without phenol red (Thermo Fisher Scientific, cat. no. 21041025)
hiPSC or hESC cell line confirmed to be free of pathogens and unwanted mutations (e.g., KOLF2.1J; The Jackson Laboratory, RRID: CVCL_B5P31)
StemFlex medium (see recipe; Thermo Fisher Scientific, cat. no. A3349401)
Dulbecco’s phosphate-buffered saline, calcium- and magnesium-free (DPBS^–^), pH range 7.0-7.3 (Thermo Fisher Scientific, cat. no. 14190250)
0.5 mM ethylenediaminetetraacetic acid [EDTA; dilute 0.5 M EDTA (Sigma-Aldrich, cat. no. 03690) 1:1000 in DPBS^–^ (see above); filter sterilize and store up to 1 year at room temperature]
StemFlex medium (see recipe) containing 10 μM Y-27632 dihydrochloride (ROCK inhibitor; see recipe)
2× freezing medium (see recipe)

6-well, 12-well, 24-well, or 10-cm plate
15- and 50-ml V-bottom polypropylene tubes (e.g., Corning Falcon)
37°C, 20% O_2_, 5%CO_2_ humidified incubator
Inverted phase-contrast microscope
Isopropanol cell-freezing container
Liquid nitrogen tank

*NOTE:* To ensure cultures are kept sterile, cells should be handled in a Class II biosafety cabinet using standard sterile technique at all times (Mather & Roberts, 1998)

1. Coat 6-well plates or 10-cm plates with a thin layer of Geltrex, a soluble reduced growth factor basement membrane extract that facilitates cell attachment.
  - Thaw Geltrex matrix on ice overnight at 4°C.
    *Rapid Geltrex thawing can cause premature gelling, resulting in uneven coating*.
  - Prepare a 1:10 Geltrex stock by diluting a 5-ml Geltrex vial (~15 mg/ml) in 45 ml ice-cold DMEM/F12 without phenol red and gently pipetting up and down to mix. During preparation, keep all reagents on ice. Aliquot and store 0.1% Geltrex stock at –20°C. Avoid freeze-thaw cycles.
  - To prepare a working solution of Geltrex for hPSC maintenance, further dilute the stock solution, either freshly prepared as in step 1b, above, or thawed at 4°C overnight, 1:10 in ice-cold DMEM/F12 without phenol red to a 1:100 final concentration (~0.15 mg/ml). Again, keep all reagents on ice during this process.
  - Add 1 ml of cold 0.01% Geltrex per well of a 6-well plate, or 5 ml per 10-cm plate, taking care to avoid bubbles.
  - To coat plates for same day use, add Geltrex solution to the wells and incubate plate at 37°C for 1 hr. To coat plates for next-day use, add Geltrex solution to the wells, wrap plates in Parafilm, and store them at 4°C up to 72 hr. Before use, incubate plate with Geltrex at 37°C for ~1 hr.
  - Before plating cells, aspirate excess Geltrex. The coated plate is ready to receive cells for plating as described in step 3h.
    *After coating the plate, take care not to let it dry out at any time. Avoid bubbles*.
2. When cultures are sparse, feed them every second day with StemFlex medium by aspirating old medium and performing a complete medium change, but switch to daily feeding as soon as cultures are 40%-50% confluent, and prepare to split them as described below. Note the morphology of the cultures (Fig. 1G), which should be uniform in appearance. Cells should have a high nuclear/cytoplasmic ratio (Fig. 1C), contain prominent nucleoli, grow in colonies with well-defined borders, and maintain cell-cell contact with other hSPCs.
  *Do not attempt hypothalamic differentiation if cultures were stressed by over-confluence or under-nutrition (yellowish medium) at any point in their culture history, or if there is evidence of cells with a morphology different from that of hPSCs. Eliminate unwanted differentiated cells by aspirating them at each feeding or by manually picking hPSC colonies as described elsewhere (International Stem Cell Initiative Consortium et al., 2010; Ludwig & Thomson, 2007; Santos et al., 2016)*.
3. When hPSCs reach 70%-80% confluence (Fig. 1A; ~4 days after previous split), split cells 1:10. Some cell lines may require lower or higher split ratios, and the time between splits can vary.
  - Preheat desired volume of 0.5 mM EDTA solution in a 37°C water bath for ~10 min.
  - Wash cells gently and briefly with room temperature DPBS^–^.
  - Add warm EDTA solution to cultures, 1 ml per well in 6-well plate or 5 ml per 10-cm plate.
  - Incubate with EDTA for 3-5 min at 37°C. After 3 min, check for cell detachment on a phase-contrast microscope. Cells should start to round up and take on a phase-bright appearance (Fig. 1D and E), but not spontaneously detach from the plate. Once cultures adopt this appearance, gently suck up and expel ~100 μl of the EDTA solution with a 1-ml (P-1000) pipet tip against the cells. They should easily dislodge and leave a small area devoid of cells. If cells do not dissociate easily, extend EDTA digestion for another minute and repeat this test.
  - Once cells can easily be detached from the plate, but before they spontaneously lift off, gently aspirate EDTA.
  - To dissociate cells, add 1 ml or 5 ml StemFlex with 10 μM Y-27632 for a well of a 6-well plate or 10-cm plate, respectively. Gently pipet this medium over the plate to detach cells, and pipet up and down several times to dissociate them to a suspension of small clumps of cells. Avoid bubbles.
  - Collect cells in a 15-ml V-bottom polypropylene tube and adjust volume with StemFlex containing 10 μM Y-27632 if necessary.
  - Plate the desired volume of this cell suspension to achieve a desired split ratio onto Geltrex-coated plates (see step 1) in StemFlex with 10 μM Y-27632, bringing the final volume to 2 ml per well of a 6-well plate or 10 ml per 10-cm plate. Culture cells in StemFlex with 10 μM Y-27632 for 1 day at 37°C, in a 20% O_2_, 5% CO_2_ humidified incubator, and then maintain them by feeding every second day with StemFlex medium.
4. Depending on experimental design, expand the culture to provide sufficient cell numbers for differentiation. Cells are ready to be plated for hypothalamic differentiation when the culture reaches ~70%-80% confluency (see Basic Protocol 2).
  *A70%-80% confluent 10-cm or 15-cm plate of hPSC yields about 8* × *10^6^ or 2* × *10^7^ cells, respectively*.
5. To cryopreserve expanded and/or excess hPSCs, 2× freezing medium should be used for long-term storage in liquid nitrogen.
  - When hPSC have reached 70%-80% confluence, dissociate cells as described above in steps 3a-3g.
  - Centrifuge collected cells in a 15-ml V-bottom polypropylene tube for 3-5 min at 160 × *g*, room temperature.
  - Aspirate supernatant and resuspend the cell pellet, adjust to desired volume (at least 1 × 10^6^ cells/ml and up to 1 × 10^7^ cells/ml).
  - Mix 0.5 ml of cell suspension with 0.5 ml of 2× freezing medium in a cryovial with a screw-top cap.
  - Place cryovials in an isopropanol cell-freezing container at room temperature, and transfer this container to -80°C overnight.
  - The following day, transfer the cryovials to a liquid nitrogen tank for long-term storage.

BASIC PROTOCOL 2

## Hypothalamic Neuron Differentiation

**Figure 2:**
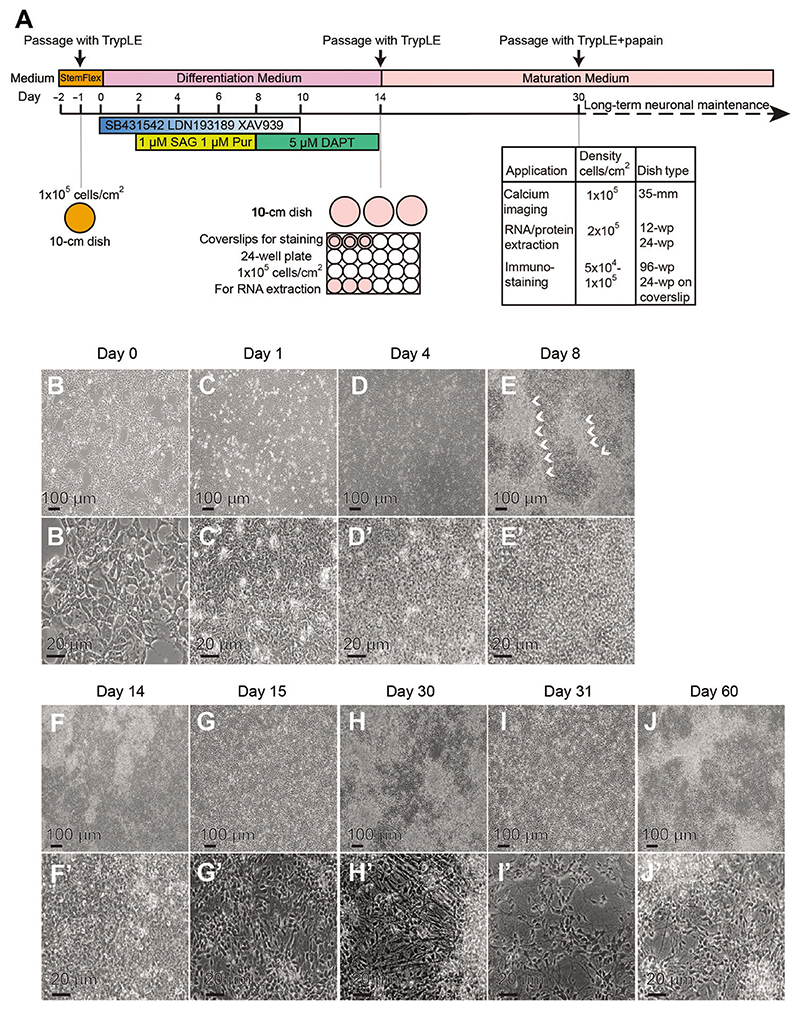
Differentiation of hPSCs to hypothalamic neurons. (**A**) Schematic of hypothalamic differentiation protocol. (**B,B**’) Dissociated hPSCs that were plated at a concentration of 1 × 10^5^ cells per cm^2^ on Day–1 should be at ~75% on Day 0. Differentiation medium (N2B27) with SMAD inhibitors (10μMSB431542, 100 nMLDN193189) and a Wnt inhibitor (2μMXAV939) is then added to induce a forebrain fate. (**C,C**’) Cultures should reach confluence and adopt a uniform appearance by Day 1. (**D,D**’) By Day 4, cell density is dramatically increased, but cells are still largely a single adherent layer. The concentrations of the SMAD inhibitors and Wnt inhibitors are progressivelyreduced over the course of differentiation, as described in Basic Protocol 2, and cultures are ventralized from Day 2 to Day 8 by adding 1 μM Smoothened agonist (SAG) and 1 μM purmorphamine (Pur). (**E,E**’) By Day 8, cultures adopt a clear neuroepithelial morphology, as evidenced by the formation of neural-ridge-like structures (arrows). At this time point, 5 μM of the NOTCH inhibitor DAPT is added until Day 14 to encourage neurogenesis. (**F,F**’) On Day 14, cultures are re-plated, at which point it is prudent to plate a fraction of the differentiated cells into a 24-well plate forqualitycontrol. After Day 14, cells are maintained in maturation medium. (**G,G**’) After passaging, most cells have a neuronal morphology. (**H,H**’) By Day 30, cultures contain a high percentage of cells with neuronal morphology but may also have some cell-dense regions. It is advisable to re-plate cultures again at this point to purify cell types of interest and decrease cell density so as to ensure that neurons receive adequate nutrients. (**I,I**’) Culture morphology at Day 31 after re-plating at 1 × 10^5^ cells per cm^2^. (**J,J**’) Cellular morphology of Day 60 cultures re-plated at 1 × 10^5^ cells per cm^2^ on Day 30. Abbreviations: 12-wp, 12-well plate; 24-wp, 24-well plate; 96-wp, 96-well plate.

The efficient generation of human hypothalamic neurons from hPSCs is based on developmental principles. The signaling pathways that lead to neuralization, forebrain specification, and ventralization are manipulated by small-molecule drugs. Specifically, the Wingless-related integration site (WNT), transforming growth factor beta (TGFβ), and bone morphogenetic protein (BMP) signaling pathways are inhibited with XAV939, LDN-193189, and SB431542, respectively, which is followed by activation of the Sonic Hedgehog (SHH) pathway with purmorphamine and Smoothed agonist (SAG). Later addition of the gamma-secretase inhibitor DAPT promotes neurogenesis by inhibiting NOTCH signaling (Fig. 2A; Merkle et al., 2015; Wang et al., 2015). The steps of this protocol are summarized in Figure 2.

This protocol is designed for adherent differentiation in monolayers. Typically, more than 1 × 10^8^ neurons are generated per 10-cm plate of hPSCs, providing large numbers of human hypothalamic neurons for functional studies, disease modeling, cellular transplantation, or drug screening. This differentiation protocol omits animal-derived products such as knockout-serum replacement (KOSR) medium and recombinant proteins in favor of chemically defined media and small-molecule drugs to ensure greater robustness and reproducibility. Note, however, that FBS is still used for cell cryopreservation. Furthermore, the resulting cells can be matured and maintained over long periods of time (at least 6 months) to enable the study of long-term developmental processes.

### Materials

Nearly confluent culture of hESCs or hiPSCs on Geltrex-coated plate (generated in Basic Protocol 1)
Dulbecco’s phosphate-buffered saline, calcium- and magnesium-free (DPBS^–^), pH range 7.0-7.3 (Thermo Fisher Scientific, cat. no. 14190250)
1× TrypLE Express, no phenol red (Life Technologies, cat. no. 12604021)
StemFlex medium containing 10 μM Y-27632 dihydrochloride (ROCK inhibitor; see recipe)
hPSC wash medium (see recipe)
0.4% trypan blue (Invitrogen, cat. no. T10282)
N2B27 medium (see recipe)
XAV939 (see recipe)
LDN-193189 (see recipe)
SB431542 (see recipe)
Smoothened agonist (SAG; see recipe)
Purmorphamine (see recipe)
DAPT (see recipe)

6-well, 12-well, 24-well, or 10-cm plate
37°C, 20% O_2_, 5%CO_2_ humidified incubator
Inverted phase-contrast microscope
15- and 50-ml V-bottom polypropylene tubes (e.g., Corning Falcon)
Laboratory centrifuge (Eppendorf 5804, rotor A-4-44, or similar)
0.5- and 1.5-ml polypropylene tubes (e.g., Eppendorf)
Cell counting slides (Countess Cell Counting Chamber Slides, Life Technologies, cat. no. C10228)
Automated cell counter and associated consumables (e.g., Life Technologies, Countess II Automated Cell Counter, cat. no. AMQAX1000)

Additional reagents and equipment for Geltrex coating of plates and culture of hPSCs (see Basic Protocol 1)

1. Coat 12-well plates or 6-well plates with Geltrex for differentiation as described above (Basic Protocol 1, step 1).
2. Dissociate and replate hPSCs (cultured in Basic Protocol 1) for differentiation.
  *Before induction of differentiation, hPSCs should lack obvious signs of differentiation or contamination, and be in a rapid growth phase*.
  - Aspirate culture medium and briefly and gently wash cell culture in roomtemperature DPBS^–^.
  - Add 37°C TrypLE to cell culture, 1 ml per well in 6-well plate or 5 ml per 10-cm plate.
  - Incubate cell culture for 3-5 min at 37°C. After a 3-min incubation, check to see if cells are detaching. Under a phase-contrast microscope, the cells should start to round up and take on a phase-bright appearance, but not spontaneously detach from the plate. Once cultures adopt this appearance, gently suck up and dispel ~100 μl of the TrypLE solution with a 1-ml (P-1000) pipet tip against the cells. They should easily dislodge and leave a small area devoid of cells. If cells do not dissociate easily, extend TrypLE digestion for another minute and repeat this test.
    *Take care to avoid over-digestion, which can cause cell death. It is also crucial that cells do not spontaneously detach in TrypLE, or excessive cell death will occur*.
  - Gently aspirate TrypLE.
  - To dissociate cells, add 1 ml or 5 ml StemFlex with 10 μM Y-27632 for a well of a 6-well plate or 10-cm plate, respectively, and gently pipet this medium over the plate to detach cells and dissociate them to a single-cell suspension.
  - Collect cells in 15-ml V-bottom polypropylene tube and adjust volume to 10 ml with StemFlex with 10 μM Y-27632.
    *This wash step dilutes residual TrypLE to slow further digestion*.
  - Centrifuge cells 3-5 min at 160 × *g*, room temperature. Aspirate supernatant, resuspend cells in 1 ml hPSC wash medium with a 1-ml (P-1000) pipet tip, and then bring to 10 ml with wash medium.
  - Centrifuge cells 3-5 min at 160 × *g*, room temperature. Aspirate supernatant and resuspend cells in 1 ml StemFlex with 10 μM Y-27632.
    *These wash steps remove any remaining traces of TrypLE*.
  - After resuspending the cell pellet, adjust volume so that the suspension is visibly turbid, but not milky (~1–5 × 10^6^ calls/ml).
  - In a 0.5-ml polypropylene tube, mix 10 μl of this cell suspension with 10 μl of 0.4% trypan blue, and then transfer 10 μl of that mixture onto cell counting slide. Count cells with automated cell counter.
  - Plate cells onto Geltrex-coated plates in StemFlex with 10 μM Y-27632, at a concentration of 1 × 10^5^ cells per cm^2^ (corresponding to 3.5 × 10^5^ cells per well of a 12-well plate or 9.5 × 10^5^ cells per well of a 6-well plate). This density corresponds to ~75%-80% confluence the following day. Ensure that cells are evenly distributed across the plate by gently shaking the plate left to right, then top to bottom, after transferring it to the incubator.
    *If cells are sparser, wait until they reach the desired density before starting the differentiation. Sparse or over-confluent cells will not pattern well*.
3. The following day, if cells plated for differentiation are evenly distributed over the plate and ~75%-80% confluence (Fig. 2B,B′), start differentiation by adding Day 0 (D0) medium (see below). Every second day, make full medium changes (5 ml per 12-well plate, 10 ml per 6-well plate) using media with the following compositions corresponding to the day of differentiation as indicated below and in Table 1. Observe cells daily for changes in morphology.
  *From Days 0-2, the culture should reach confluence and cells should have a simple and uniform hPSC-like morphology (Fig. 2C,C′). By Day 4, cultures are highly compacted and adopt a more rounded appearance (Fig. 2D,D′). Between Days 4 and 8, the cultures take on a dense neuroepithelial morphology with identifiable neural ridgelike structures. (Fig. 2E,E′). A neuroepithelial morphology is still evident before passaging on Day 14 (Fig. 2F,F′). For issues that can arise during the differentiation process, see* Troubleshooting.
  Day 0 (D0): N2B27 + 2 μM XAV939 + 100 nM LDN-193189 + 10 μM SB431542
  Day 2 (D2): N2B27 + 2 μM XAV939 + 100 nM LDN-193189 + 10 μM SB431542 + 1 μM SAG + 1 μM purmorphamine
  Day 4 (D4): N2B27 + 1.5 μM XAV939 + 75 nM LDN-193189 + 7.5 μM SB431542 + 1 μM SAG + 1 μM purmorphamine
  Day 6 (D6): N2B27 + 1 μM XAV939 + 50 nM LDN-193189 + 5 μM SB431542 + 1 μM SAG + 1 μM purmorphamine
  Day 8 (D8): N2B27 + 0.5 μM XAV939 + 25 nM LDN-193189 + 2.5 μM SB431542 + 5 μMDAPT
  Day 10 (D10): N2B27 + 5 μMDAPT
  Day 12 (D12): N2B27 + 5 μMDAPT
4. On Day 14 (D14), dissociate and cryopreserve cultures for later replating (see Support Protocol 2), or replate them straight away (see Basic Protocol 3).

***SUPPORT PROTOCOL 1***

## Cortical Neuron (Control) Differentiation

In order to confirm correct and efficient hypothalamic patterning of hPSCs, it is helpful to compare hypothalamic differentiation to a parallel differentiation of hPSCs to cortical neurons (Shi et al., 2012). To facilitate parallel culture, the time course and basic procedure of this differentiation is similar to that for hypothalamic differentiation, except that ventralizing factors (SAG and purmorphamine) are omitted, and DAPT is replaced with FGF2.

### Additional Materials (also see Basic Protocol 2)

FGF2 (see recipe)

1. Follow steps 1 and 2 of Basic Protocol 2.
2. Feed cells using the volumes and timing given in Basic Protocol 2, step 3, but use the following supplements instead (see also Table 2).
  Day 0 (D0): N2B27 + 2 μM XAV939 + 100 nM LDN-193189 + 10 μM SB431542
  Day 2 (D2): N2B27 + 2 μM XAV939 + 100 nM LDN-193189 + 10 μM SB431542
  Day 4 (D4): N2B27 + 1.5 μM XAV939 + 75 nM LDN-193189 + 7.5 μM SB431542
  Day 6 (D6): N2B27 + 1 μM XAV939 + 50 nM LDN-193189 + 5 μM SB431542
  Day 8 (D8): N2B27 + 0.5 μM XAV939 + 25 nM LDN-193189 + 2.5 μM SB431542
  Day 10 (D10): N2B27
  Day 12 (D12): N2B27 + 20 ng/ml FGF2
3. On Day 14 (D14), dissociate and cryopreserve cultures for later replating (see Support Protocol 2), or replate them straight away (see Basic Protocol 3).

***BASIC PROTOCOL 3***

## Neuronal Maturation

The aim of this protocol is to facilitate the maturation of neurons to the point that they are spontaneously electrically active and respond to exogenous factors, such as hormones, drugs, and metabolites that their counterparts in the brain might also respond to. To promote maturation and induction of target genes such as *POMC*, cultures are re-plated at 3 × 10^5^ cells per cm^2^, and 10 ng/ml brain-derived neurotrophic factor (BDNF) is added to an enhanced maturation medium, Synaptojuice or SJ (Chen et al., 2021; Telezhkin et al., 2016). Hypothalamic neurons begin to acquire functional properties after 30 days of culture and mature further over subsequent weeks in culture. Plating dissociated human neurons on a monolayer of glia (3 × 10^4^ cells per cm^2^) harvested from the newborn mouse or rat brain or derived from hPSCs may accelerate functional maturation and may be particularly valuable for experiments in which neurons are plated at low densities or are to be used for electrophysiological recording (Ullian et al., 2001; Zuchero & Barres, 2015). Protocols for glial isolation and culture are described elsewhere (Albuquerque et al., 2009; Foo et al., 2011). The same maturation protocol can be used for hypothalamic and cortical differentiation. Please note that maturation can proceed immediately from differentiation (Basic Protocol 2 or Support Protocol 1) or upon thawing cryopreserved progenitors (Support Protocol 2).

### Materials

Dulbecco’s phosphate-buffered saline, calcium- and magnesium-free (DPBS^–^), pH range 7.0-7.3 (Thermo Fisher Scientific, cat. no. 14190250)
Neural progenitor cells after differentiation (Basic Protocol 2)
1× TrypLE Express, no phenol red (Life Technologies, cat. no. 12604021)
Trituration medium (see recipe)
0.4% trypan blue (Invitrogen, cat. no. AM7962)
Maturation medium (see recipe)
Maturation medium (see recipe) containing 10 μM Y-27632 dihydrochloride (ROCK inhibitor; see recipe)
Enhanced maturation medium: Synaptojuice 1 (SJ1; see recipe)
BDNF (see recipe)
Enhanced maturation medium: Synaptojuice 2 (SJ2; see recipe)
Papain (Worthington Biochemical Corporation, cat. no. LK003176)

6-well, 12-well, 24-well, or 10-cm plate
5- or 10-ml serological pipets (stripettes) and pipet holder
P1000 pipet and tips
37°C, 20% O_2_, 5%CO_2_ humidified incubator
Inverted phase-contrast microscope
15-ml and/or 50-ml V-bottom polypropylene tubes (e.g., Corning Falcon)
Laboratory centrifuge (Eppendorf 5804, rotor A-4-44, or similar)
0.5- and /or 1.5-ml polypropylene tubes (e.g., Eppendorf)
Automated cell counter and associated consumables (e.g., Life Technologies, Countess II Automated Cell Counter, cat. no. AMQAX1000)
Cell counting slide (Countess Cell Counting Chamber Slides, cat. no. C10228)

Additional reagents and equipment for coating plates with Geltrex (Basic Protocol 1) and differentiation of neurons (Basic Protocol 2 or Support Protocol 1)

1. Coat plates with Geltrex for differentiation as described in Basic Protocol 1, step 1, except use a 1:50 (~0.3 mg/ml) final Geltrex concentration to facilitate neuronal attachment.
2. On Day 14, neural progenitors generated via Basic Protocol 2 or Support Protocol 1 are dissociated and re-plated to encourage neurogenesis and neuronal survival and maturation.
  - Wash cell culture gently in DPBS^–^.
  - Add TrypLE to cells, 1 ml per well in 6-well plate or 5 ml per 10-cm plate.
  - Incubate cell culture for 3-5 min at 37°C. After 3 min of incubation, check to see if cells are detaching. Under a phase-contrast microscope, the cells should start to round up and take on a phase-bright appearance, but not spontaneously detach from the plate. Once cultures adopt this appearance, gently suck up and dispel ~100 μl of the TrypLE solution with a 1-ml (P-1000) pipet tip against the cells. They should easily dislodge and leave a small area devoid of cells. If cells do not dissociate easily, extend TrypLE digestion for another minute and repeat this test.
    *Take care to avoid over-digestion, which can cause cell death and release of genomic DNA. It is also crucial that cells do not spontaneously detach in TrypLE, or excessive cell death will occur*.
  - Gently aspirate TrypLE, leaving the digested cells still lightly stuck onto the plate.
  - To dissociate cells, add 1 ml trituration medium per well of a 6-well plate with a P1000 gently pipet this medium over the plate to detach cells and dissociate them to a single-cell suspension. Cells should detach easily, requiring only a few passes with the P1000. For a 10-cm plate, use 5 ml of trituration medium with a 5- or 10-ml stripette
  - Collect cells in a 15-ml V-bottom polypropylene tube and adjust volume to 10 ml with trituration medium.
  - Centrifuge cells 3-5 min at 160 × *g*, room temperature. Aspirate supernatant and resuspend cells in 10 ml trituration medium.
  - After resuspending the cell pellet, adjust volume so that the suspension is visibly turbid, but not milky (~1-5 million cells/ml).
  - In two separate polypropylene tubes, mix 10 μl ofthis cell suspensionwith10 μl of 0.4% trypan blue, transfer 10 μl of that mixture onto cell counting slide, and count cells with automated cell counter. Ensure that the two counts are similar and then take the average of them to calculate cell number.
    *To achieve accurate counts, ensure that the cell suspension has just been gently but thoroughly agitated prior to taking an aliquot for counting*.
  - Centrifuge cells 3-5 min at 160 × *g*, room temperature. Aspirate supernatant, and then resuspend cells in a desired volume of maturation medium with 10 μM Y-27632 (ROCK inhibitor) to enable plating at the desired density as described below, or cryopreservation as described in Support Protocol 2.
3. Plate cells onto Geltrex-coated plates in maturation medium with 10 μM Y-27632 at a concentration of 3 × 10^5^ cells per cm^2^ (corresponding to 1.05 × 10^6^ cells per well of a 12-well plate and 2.88 × 10^6^ cells per well of a 6-well plate).
  *This usually corresponds to 1:3 passage ratio, but exact figures can vary*.
4. Maintain cultures as follows:
  - On the day after plating (Day 15), aspirate medium to remove Y-27632 and gently feed with a standard volume of maturation medium (e.g., 2 ml per well of a 6-well plate).
  - On Day 16, aspirate medium and add twice the normal volume of SJ1 for neuronal maintenance (e.g., 4 ml per well of a 6-well plate).
    *This larger volume helps ensure that neurons are exposed to a relatively constant supply of nutrients*.
  - Change 75% of medium volume (e.g., for 4 ml of medium in a 6-well plate, replace 3 ml) every second day for 3 feedings (1 week total time in SJ1). Add BDNF fresh at each feeding.
  - After 1 week in SJ1, aspirate medium and add SJ2. As described above, change 75% of medium volume every second day and add BDNF fresh at each feeding.
    *Optional: Laminin (final concentration: 1* μ*g/ml; Merck, cat. no. L2020) may added as a supplement to provide better cell attachment during the maturation period*.
5. If desired, neurons can be re-plated again at later time points to move them onto different plates for imaging or analysis. To do so, repeat the dissociation procedure outlined in steps 2 and 3 with the following exceptions: (1) resuspend 1 vial of papain per 10 ml of TrypLE at step 2b to aid in neuronal dissociation and ensure significantly higher survival upon re-plating, and (2) when re-plating cells, use SJ2 medium supplemented with 10 μM Y-27632 rather than maturation medium and maintain the cells in SJ2 using the feeding schedule, volume, and supplements as described in step 4.
  *Depending on experiments and if long-term culture is desired, consider culturing cells at lower density* (2 × *10^4^ cell per cm^2^) on a monolayer of astrocytes plated on Geltrex-coated or poly-d-lysine-coated plastic. Non-neuronal cells may continue to proliferate, which can lead to culture overgrowth. Although cultures can be maintained for long periods of time, many experiments can be executed between Day 30 and Day 80. If non-adherent (e.g., organoid or assembloid) cultures are desired, dissociated cells can be encouraged to generate these in low-attachment round-bottom plates either at either the first passaging (D14) or later passaging steps*.

***SUPPORT PROTOCOL 2***

## Cryopreservation and Thawing of Neuronal Progenitors

Upon differentiation, neuronal progenitors can be cryopreserved for later thawing and maturation. Freezing progenitors has several advantages. First, it reduces the time at which more mature neurons can be generated for functional experiments because it cuts out the time required to thaw, expand, and passage the hPSC line (~1 week) and the time needed for differentiation (2 weeks). Second, it allows users to generate a large number of frozen vials of progenitors from a single differentiation in order to ensure more consistent results from experiment to experiment once progenitors are thawed and matured into neurons. Cryopreservation is performed once progenitors have been patterned to a specific spatial identity, but before they have given rise to neurons that are sensitive to freezing and thawing. Although there is some loss of viability upon thawing, cultures generated from cryopreserved vials have a similar composition to those generated by direct replating.

### Materials

2× freezing medium
Maturation medium (see recipe)
Y-27632 dihydrochloride (ROCK inhibitor; see recipe)

Sterile screw-top 2-ml cryovials
Label printers and cryo labels

Mr. Frosty or other freezing container capable of cooling cells at a rate of −1°C per min

1. If cultures are to be frozen down, first label the desired number of screw-top cryovials using a label maker (not by hand) with your initials, the date, the cell line name, and the passage number, leaving space to write in the cell number. Move the cryovials to a rack within the tissue culture safety cabinet (hood) that you will work in.
2. On Day 14, neural progenitors generated via Basic Protocol 2 or Support Protocol 1 are dissociated as described in Basic Protocol 3, steps 1 and 2. The resulting suspension of progenitors in maturation medium with 10 μM Y-27632 is then cryopreserved as follows:
  - Slowly but steadily over the course of 60 s under steady mixing (by pipetting a fraction of the volume at a time and/or swirling if the volume of the tube allows), add an equal volume of ice-cold 2× freezing medium to the cell suspension.
  - Working quickly to minimize the amount of time cells are exposed to DMSO, pipet 1 ml of the cell suspension into each cryovial, and rapidly cap the vials, taking care to keep the lids sterile.
  - Immediately transfer cryovials to a room-temperature Mr. Frosty or similar device to ensure even freezing, and then immediately move this device to a -80°C freezer to freeze overnight.
  - The next day, transfer frozen vials to liquid nitrogen for long-term storage.
3. To thaw frozen cryovials of progenitors, first prepare plates (see Basic Protocol 3, step 1) and warm up a sufficient volume of maturation medium with 10 μM Y-27632 (see recipe) to 37°C. Then proceed with thawing as follows:
  - Thaw cryovials in a 37°C water bath until the freezing medium containing the cell suspension has started to thaw but a portion of the cells remain frozen.
  - Quickly and gently transfer the cells to 10 ml of warm maturation medium with 10 μM Y27362, centrifuge for 3 min at 160 × *g*, room temperature, and aspirate the supernatant.
  - Resuspend the cell pellet in 10 ml medium as above.
  - Repeat step b to remove any traces of DMSO, resuspend cell pellet in the desired volume of warm maturation medium with 10 μM Y27362, and proceed with plating as described in Basic Protocol 3, step 3.

***SUPPORT PROTOCOL 3***

## Quality Control: Confirmation of Hypothalamic Patterning and Neurogenesis

**Figure 3:**
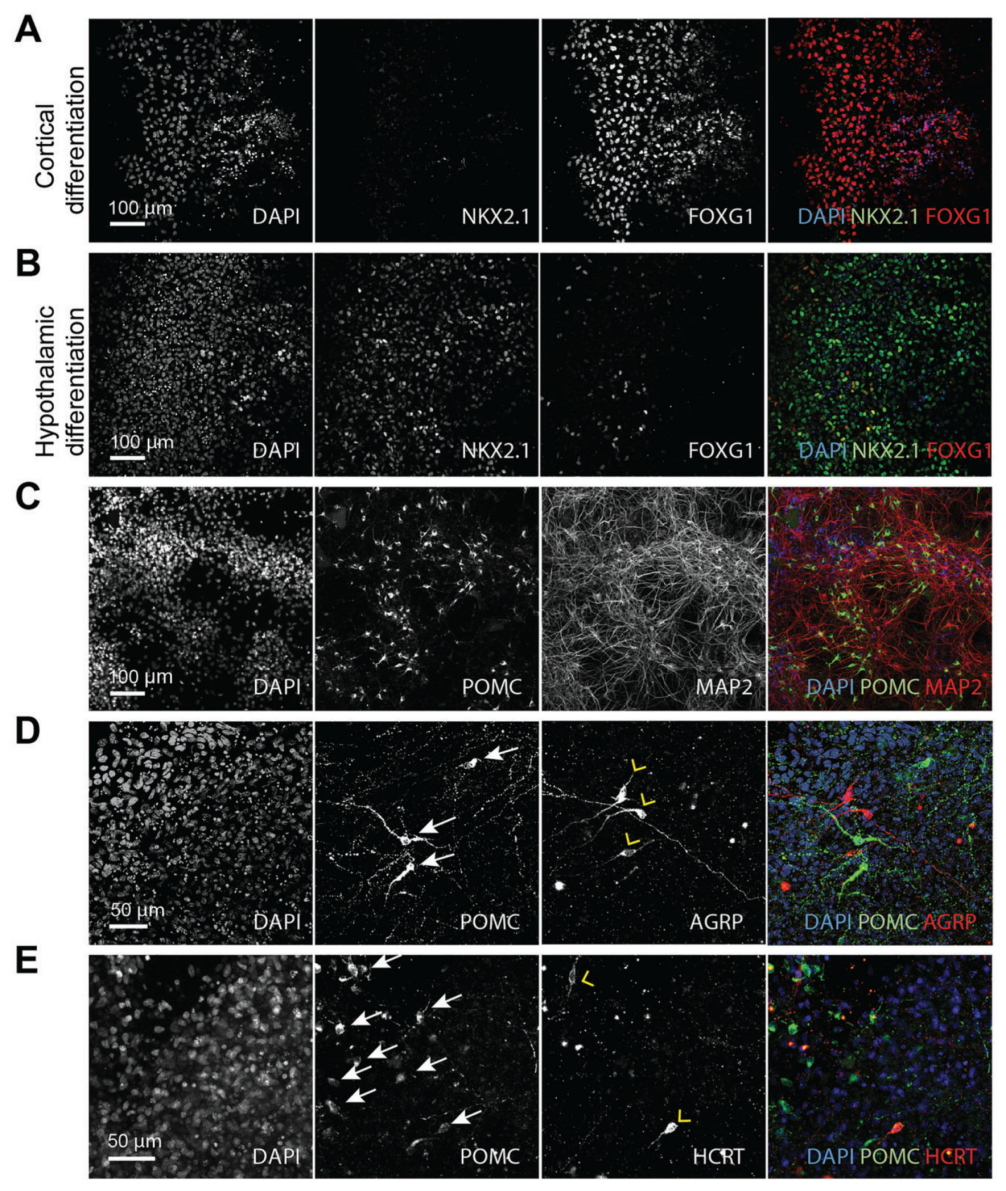
Quality control of hPSC hypothalamic differentiations by immunocytochemistry. (**A**) Immunofluorescence staining on Day 10 of hES cells differentiated to dorsal forebrain (cortical) cells. The vast majority of cells in these cultures express the transcription factor FOXG1, which is expressed in the telencephalon in vivo, and a few cells express the transcription factor NKX2.1, which is expressed in the ventral forebrain. DNA is stained with DAPI to visualize cell nuclei. (**B**)OnDay 10 of hypothalamic differentiation, the vast majority of cells express NKX2.1 and only a few express FOXG1. (**C**) On Day 24 of hypothalamic differentiation, some hPSC-derived cells express POMC and many cells express microtubule-associated protein 2 (MAP2), a dendritic marker indicative of neuronal identity. (**D and E**) On Day 119 of differentiation, hypothalamic cultures retain numerous neurons expressing POMC (arrows), Agouti-related peptide (AGRP; arrowheads), and hypocretin/orexin (HCRT; arrowheads).

To confirm successful hypothalamic patterning and generation of hypothalamic neurons, we recommend performing quality-control experiments based on immunostaining and RT-qPCR before performing further experiments. Hypothalamic patterning can be gauged by the expression of regionally expressed transcription factors (Fig. 3A and B), and hypothalamic neurogenesis can be confirmed by testing for neuropeptides that are highly enriched in the hypothalamus (Fig. 3C, D, and F; Tables 3–5).

*CAUTION:* Wear appropriate personal protective equipment and ensure that you are familiar with Material Safety Data Sheet before working with concentrated hydrochloric acid. Also, take appropriate precautions when handling sodium azide, which is highly toxic.

### Materials

5 M hydrochloric acid (HCl; Thermo Fisher, cat. no. MFCD00011324–2.5 L] 70% (v/v) ethanol
Differentiated neuronal culture (Basic Protocol 3, step 2, substeps a–l)
Dulbecco’s phosphate-buffered saline, calcium- and magnesium-free (DPBS^–^), pH range 7.0-7.3 (Thermo Fisher Scientific, cat. no. 14190250)
4% paraformaldehyde in PBS (Santa Cruz, cat. no. 30525-89-4, or Alfa Aesar, cat. no. J61899)
Primary antibodies (Table 4)
Tris-buffered saline (TBS; see recipe)
Normal donkey serum (Jackson Immunoresearch Labs, cat. no. 017-000-121)
Triton X-100 (Sigma-Aldrich, cat. no. T8787)
Alexa Fluor-conjugated secondary antibody (Thermo Fisher Scientific; conjugated to Alexa Fluor 488, 555, 594, or 647)
4′,6-Diamidine-2′-phenylindole dihydrochloride (DAPI; see recipe)
Sodium azide (Sigma-Aldrich, cat. no. 769320)
ProLong Diamond Antifade Mountant (Thermo Fisher Scientific, cat. no. P36965)
RNA isolation kit (RNeasy Mini Kit; Qiagen, cat. no. 74104)
Human brain total RNA (Thermo Fisher Scientific, cat. no. AM7962)

Glass coverslips (Thermo Scientific, cat. no. MENZCB00120RAC20)
65°C rolling or shaking incubator
Glass beaker
6-well, 12-well, 24-well, or 10-cm plate
Forceps
Glass microscope slides

Additional reagents and equipment for Geltrex coating of plates (Basic Protocol 1, step 1) and RT-qPCR (Toyohara et al., 2015)

1. Prepare coverslips for cell plating.
  - Incubate glass coverslips in 5 M HCl at 65°C overnight, preferably in a rolling or shaking incubator to enable even acid washing of coverslips.
    CAUTION: *Wear appropriate personal protective equipment and ensure that you are familiar with Material Safety Data Sheet before working with concentrated acid*.
  - Carefully remove acid and appropriately dispose of HCl or store safely for later use, and transfer coverslips to a clean glass beaker.
  - Thoroughly wash coverslips five times with large volumes of distilled water.
  - Cover and store coverslips in 70% ethanol. Cover with Parafilm and store at room temperature indefinitely
  - In a class II biosafety cabinet, transfer one acid-washed and ethanol-sterilized glass coverslip per well of a 24-well plate using a sterile pair of forceps. Transfer a sufficient number of coverslips to analyze the desired number of antibodies for each cell line and differentiation protocol, keeping in mind biological and technical replicates.
  - Once coverslips have completely dried, coat them with Geltrex as described in Basic Protocol 1, step 1.
    *At this point, it is useful to coat a sufficient number of wells with Geltrex for RT-qPCR analysis, as described below, if this analysis is to be carried out, especially for analysis at later time points such as Day 40*.
  - Plate differentiated neurons onto 24-well plate with a Geltrex-coated glass coverslip in each well at a density of ~1 × 10^5^ cells/cm^2^.
  - Allow cells to recover for 24-48 hr.
  - Gently aspirate medium and wash cells with DPBS^–^, taking care to avoid cell detachment.
  - In a chemical hood, gently add 4% paraformaldehyde (PFA) and incubate cells 10 min at room temperature.
    *If using PFA from a frozen stock, ensure that PFA is completely thawed but not warm*.
  - In the chemical hood, collect PFA for waste disposal, but take care not to let cells dry.
  - Wash cells three times with TBS, again taking care not to let cells dry.
    *To ensure gentle washing of cells, it is advisable to use a plastic transfer pipet capped with a 200-μl (P-200) pipet tip*.
    *Fixed cells can be stored in TBS at this point at 4°C for up to 72 hr before staining*.
2. Stain cells.
  - After fixing cells, remove TBS and add primary antibody at the appropriate dilution (Table 4) in TBS containing 10% normal donkey serum plus 0.1% Triton X-100 to fixed cells; incubate cells at 4°C overnight.
  - Wash cells three times briefly and gently with TBS, and then once for at least 30 min.
  - Add secondary antibody solution diluted 1:500 in TBS containing 10% normal donkey serum plus 0.1% Triton X-100, and incubate cells 2 hr at room temperature.
  - Wash cells with TBS three times briefly and gently, and then once for at least 30 min.
  - Add 360 nM DAPI solution and incubate cells 5 min at room temperature in darkness.
  - Wash cells three times with TBS.
  - Store plates at 4°C in TBS with 0.1% sodium azide, protected from light.
    *If cells are on coverslips, mount them onto glass slides as described below. If cells are on plates, add an appropriate volume of TBS with 0.1% sodium azide for the size of the well and seal the plate with adhesive film to prevent evaporation*.
    CAUTION: *Please take appropriate precautions when handling sodium azide, which is highly toxic*.
3. Mount slides.
  - Add 1 drop of mounting medium (ProLong Diamond Antifade Mountant) onto a glass slide, taking care to avoid introducing air bubbles.
  - Remove excess liquid from coverslip by dabbing the edge on a paper towel.
  - Place coverslip sample-side-down onto mounting medium on the glass slide.
  - Cure for 24 hr at room temperature, protected from light.
  - Gently clean coverslips with 70% ethanol and image.
4. Analyze cells by RT-qPCR
  - Collect ~1 × 10^6^ cells per condition from freshly dissociated cells or from adherent cultures dissociated with TrypLE as described above.
  - Wash and pellet cells as described above.
  - Carefully aspirate all supernatant from the cell pellet and resuspend it in 350 μl RLT buffer from the Qiagen RNeasy Mini Kit.
  - Isolate RNA according to manufacturer’s instruction using the Qiagen RNeasy Mini Kit.
  - Analyze gene expression by TaqMan (see Table 5) RT-qPCR as described elsewhere (Toyohara et al., 2015). As a positive control, we use commercially available human whole brain RNA.

***SUPPORT PROTOCOL 4***

## Bulk RNA Sequencing of Hypothalamic Cultures

Bulk RNA sequencing can be performed on hPSC-derived hypothalamic cultures after treatment with pertinent drugs or compounds to study the transcriptomic response. This can be performed on the entire culture, or on a specific sub-population identified by virtue of a reporter. We describe here the transcriptomic analysis of differentiated and matured hypothalamic neurons generated from a fluorescent reporter cell line and purified by fluorescence-activated cell sorting (FACS) from the heterogeneous culture following drug treatment. The sorted cells can then be processed for RNA extraction and generation of cDNA libraries with low RNA input range of 250 pg to 10 ng suitable for next-generation sequencing.

### Materials

Dulbecco’s phosphate-buffered saline, calcium- and magnesium-free (DPBS^–^), pH range 7.0-7.3 (Thermo Fisher Scientific, cat. no. 14190250)
hPSC-derived hypothalamic neurons from a fluorescent (e.g., GFP^+^) reporter line for a cell type of interest at Day 40+ (see Basic Protocol 3)
1× TrypLE Express, no phenol red (Life Technologies, cat. no. 12604021)
Papain (Worthington Biochemical Corporation, cat. no. LK003176)
FACS wash buffer (see recipe)
FACS sorting buffer (see recipe)
DAPI
RNeasy Micro kit (Qiagen, cat. no. 74004)
SMARTer® Stranded Total RNA-seq kit v3 - Pico Input Mammalian (Takara, cat. no. 634485)

Laboratory centrifuge (Eppendorf 5804, rotor A-4-44, or similar)
1.5- or 2-ml DNA Lo-bind polypropylene tubes (e.g., Eppendorf)
Flowmi® cell strainer (Merck, cat. no. BAH136800040)
FACS apparatus

1. Prepare treated cells for FACS sorting
  - Wash cell culture gently in DPBS^–^.
  - Resuspend 1 vial of papain per 10 ml of TrypLE. Add TrypLE + papain to cells, 1 ml per well in 12-well plate or 2 ml per well in 6-well plate.
  - Incubate cell culture for 3-5 min at 37°C. After 3 min of incubation, check to see if cells are detaching.
  - Gently aspirate TrypLE, leaving the digested cells still lightly stuck onto the plate.
  - To dissociate cells, add 1 ml or 1.5 ml FACS wash buffer per well of a 12-well or 6-well plate, respectively, and gently pipet this medium over the plate to detach cells and dissociate them to a single-cell suspension. Cells should detach easily, requiring only a few passes with the P1000.
  - Collect cells in a 1.5- or 2-ml DNA Lo-bind Eppendorf tube.
  - Centrifuge cells 3-5 min at 160 × *g*, room temperature. Aspirate supernatant and resuspend cells in FACS wash buffer.
  - Centrifuge cells 3-5 min at 160 × *g*, room temperature.
  - Aspirate supernatant and add 500 μl FACS sorting buffer.
  - Take cells to FACS machine on ice (sorting at 4°C is ideal). Before loading the cells to the machine, filter cells through a 40-μm Flowmi® cell strainer and add DAPI to a final concentration of 0.1 μg/ml to gate for live/dead cells
  - Sort the targeted GFP-positive cells directly into 350 μl of RLT lysis buffer (from the Qiagen RNeasy micro kit) and snap freeze on dry ice.
    *Note that single-cell methods such as SMART-seq can be employed to profile more rare cell populations if there is insufficient material for bulk analysis. In this case, follow steps a-j but sort individual cells, following manufacturer’s protocol*.
2. Extract RNA from sorted cells using the Qiagen RNeasy micro kit as per manufacturer’s instructions.
3. Generate cDNA libraries using commercial kits such as the SMARTer® Stranded Total RNA-seq kit v3 - Pico Input Mammalian as per manufacturer’s instructions.
4. Sequence the generated cDNA library on a chosen sequencing platform to the desired depth and requirements, such as 20 million mapped reads per sample on an Illumina sequencer.

## Calcium Imaging of Hypothalamic Cultures

One way to assess the electrical properties and functional responsiveness of hPSC-derived hypothalamic neurons is to perform calcium imaging, in which the fluorescence intensity of a calcium indicator dye, or a genetically encoded calcium indicator such as GCaMP, changes in response to the intracellular calcium concentration. Because the firing of action potentials leads to cellular depolarization and the opening of voltage-gated calcium channels, calcium imaging is frequently used to gain insight into the electrical activity of a neuron (Shuttleworth & Smith, 1999). Furthermore, the intracellular calcium concentration can change due to calcium release from intracellular stores (e.g., from the endoplasmic reticulum) and through channels regulated by signaling pathways (Grien-berger & Konnerth, 2012). For example, transient receptor potential (TRP) channels can be phosphorylated by protein kinase A (PKA) and protein kinase C (PKC) in response to G-protein-coupled receptor (GPCR) activation to increase the entry of calcium and other positively charged ions (Veldhuis et al., 2015). Calcium imaging can therefore also shed light on the activity of signal transduction pathways under baseline conditions or in response to an introduced agent (e.g., drug, hormone, peptide, or metabolite). Compared to conventional whole-cell electrophysiology, calcium imaging enables the analysis of larger cell numbers in a shorter amount of time, albeit with lower temporal precision and fewer insights into cellular membrane properties due to the much slower time frame of calcium entry and binding to indicators relative to the time course of an action potential. Although a number of different calcium indicators are available (Ikegaya et al., 2005; Russell, 2011), we describe here the use of two distinct chemical-based calcium indicators, Fura-2 AM (Basic Protocol 4) and Rhod-3 AM (Alternate Protocol). The acetoxymethyl (AM) modification of these indicators mask the negative charge of the indicators to allow them to readily enter cells by passing through the plasma membrane.

***BASIC PROTOCOL 4***

## Calcium Imaging of Hypothalamic Neurons Using Fura-2 Am

Fura-2 AM is a ratiometric indicator that fluoresces more brightly at 340 nm and more weakly at 380 nm in the presence of calcium, enabling the 340 nm/380 nm fluorescence intensity ratio of Fura-2AM to indicate changes in intracellular calcium levels (Takahashi et al., 1999). Using this ratio, rather than absolute fluorescence intensity, minimizes the effects of photobleaching and other technical artifacts such as uneven dye loading into cells. In cultures ~30 days old, calcium imaging can be used to investigate how the hPSC-derived neurons respond to stimuli. Neurons older than Day 50 tend to be more active and are better suited to studying synaptic activity (Kirwan et al., 2015; Prè et al., 2014; Shi et al., 2012).

*NOTE:* For the fluorescence imaging system, a number of systems can be used, and the configuration below serves as a guide. Many users will prefer an inverted microscope setting, although an upright configuration that facilitates electrophysiological recording is also possible. Importantly, the illumination and filters chosen need to be suitable for the intended fluorophores, the objective needs to be of sufficient magnification and numerical aperture (NA) to efficiently collect emitted photons, and the camera should be fast and sensitive enough to detect these photons. Software to control shutters and collect movies that can then be analyzed in R or similar analysis software is also key.

## Materials

35-mm Ibidi dishes (Thistle Scientific, cat. no. 80136), coated with Geltrex (see Basic Protocol 1, step 1)
hPSC-derived hypothalamic neurons, Day 30+ (preferably Day 40-60; see Basic Protocol 3)
BrainPhys™ medium (Bardy et al., 2015; STEMCELL Technologies, cat. no. 05790)
Recording solution: Artificial cerebrospinal fluid (ACSF; see recipe), or Hank’s balanced salt solution no phenol red (HBSS, cat. no. 14025092; Thermo Fisher scientific), or BrainPhys™ Imaging optimized neuronal medium (Zabolocki et al., 2020; STEMCELL Technologies, cat. no. 05796), prewarmed to 37°C
Fura-2AM loading solution (see recipe)
ACSF with 30 mM KCl (see recipe), or BrainPhys™ Imaging medium with 30 mM KCl, or HBSS with 30 mM KCl

37°C, 20% O_2_, 5%CO_2_ humidified incubator
Perfusion system: we use a custom-built gravity perfusion system, but commercial systems are also available, e.g., MPS-2 (World Precision Instruments)
Inverted phase-contrast microscope
Fluorescence imaging system:
  Fluorescence microscope, e.g., Eclipse TE2000U (Nikon)
  Epifluorescence illuminator and monochromator to provide excitation wavelengths at 340 nm, 360 nm, 380 nm (e.g., Optoscan; Cairn Research Ltd.)
  Emission filter: 510 nm
  Objective: 40× Nikon oil immersion, NA 1.3
  CCD camera, e.g., Orca-ER-1394 (Hamamatsu)
Imaging software: e.g., Metafluor (Molecular Devices, cat. no. RRID: SCR_014294)

Additional reagents and equipment for Geltrex coating (Basic Protocol 1, step 1; note that laminin and other coatings can also be used to support neuronal adhesion for calcium imaging depending on the user’s preference)

**Figure 4:**
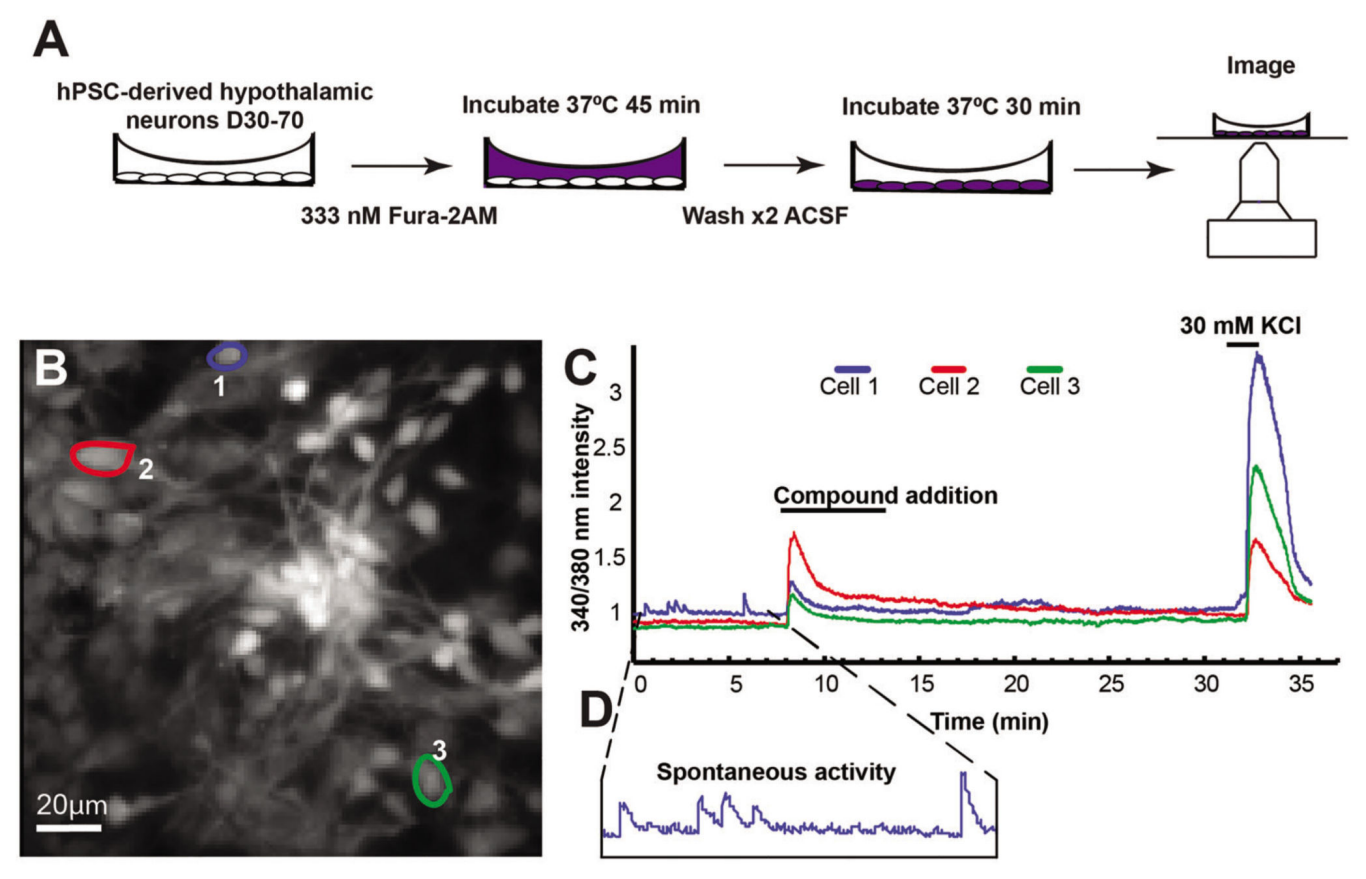
Calcium imaging of hPSC-derived hypothalamic neurons. (**A**) Schematic showing Fura-2AM loading of neurons. Cells are incubated in 333 nM Fura-2AM for 45 min in an incubator at 37°C. After cultures are gently washed twice with the chosen recording solution (ACSF in this case), cells are incubated for a further 30 min before imaging is performed. (**B**) A typical field of Fura-2AM-loaded hypothalamic neurons at Day 77 post differentiation, with cells of interest highlighted (1, 2, and 3). (**C**) Trace of the 340 nm/380 nm intensity ratio over time in the three cells highlighted in **B**, showing a depolarization in the cells after perfusion of an exogenous compound and a large depolarization upon perfusion with ACSF containing 30 mM potassium chloride (KCl). (**D**) Spontaneous calcium transients in the absence of stimulation.

1. Prepare cells for calcium imaging with Fura-2 AM.
  - Seed neurons at 1-1.5 × 10^5^ cells per cm^2^ onto Geltrex-coated 35-mm Ibidi dishes (see Basic Protocol 3, step 2, substep o) in SJ2.
  - Maintain the cells in a tissue culture incubator at 37°C with 5% CO_2_.
    *To detect spontaneous and synaptic activity, it is recommended that cells be allowed to recover for at least 1 week after plating*.
  - At 24-48 hr before imaging, replace SJ2 with warm (37°C) BrainPhys™ neuronal medium and maintain the cells in a tissue culture incubator at 37°C with 5% CO_2_.
    *BrainPhys™ medium (BP; Bardy et al., 2015) promotes neuronal activity and maturity of hPSC-derived neurons*.
  - Transfer cells to warm (37°C) ACSF (or any other chosen recording solution such as HBSS or BPI) containing 1 ml of Fura-2AM loading solution per 35-mm dish (Fig. 4A).
    *BrainPhys™ imaging medium (BPI; Zabolocki et al., 2020) is optimized to improve the quality of live imaging of neurons in vitro by reducing phototoxicity and by maintaining the neuronal physiological activity*.
  - Transfer cells to a 37°C, 5% CO_2_ tissue culture incubator for 45 min.
  - Remove loading solution from the cells and gently wash cultures twice with warm (37°C) recording medium.
  - Transfer cells to a tissue culture incubator for a further 30 min in 1 ml (per 35-mm dish) of warm (37°C) recording medium before imaging.
2. Perform calcium imaging.
  *If the goal of the experiment is to record intrinsic calcium responses from individual neurons, it is recommended that the neuron under investigation be pharmacologically uncoupled from the neuronal network by adding synaptic blockers on the extracellular recording solution (see recipe). If studying synaptic activity, it is recommended that cells be recorded at 37°C in a synaptic-blocker-free extracellular bath solution. To maintain the cells at 37°C for the entire length of the experiments, use a heated bath chamber (e.g., PTC Mini Chamber III, Luigs & Neumann) connected to a temperature controller (e.g., Temperature controller TC07, Luigs & Neumann is recommended)*.
  - If perfusion is required, prepare perfusion system during the final incubation after loading the cells with Fura-2AM. Set it up so that perfusion flows at a rate of ~3 ml/min. Turn on heating if required.
  - Transfer cells to imaging system and begin perfusion with recording solution. Allow 10 min for perfusion and temperature to stabilize.
  - Check for fluorescence under UV illumination.
  - Navigate to a field where the cell density allows clear resolution of single cells and that also enables the measurement of background intensity (Fig. 4B)
  - Use software to set up acquisition at 340-, 360-, and 380-nm excitation, and detection of emitted light at 515 nm.
    *The 360-nm channel is used to determine the extent to which photobleaching is occurring. This is done by comparing the first and last image in the 360-nm channel from the dataset and checking whether fluorescence intensity was lost during the experiment*.
  - Focus the cells and adjust the gain and exposure settings so that the signal is not saturated. We recommend exposures of 50-200 ms to minimize bleaching.
  - Use the imaging software to highlight cell bodies of interest and a background area. Use the background area to perform a background signal subtraction.
    *Observations of neuronal activity and background subtraction can also be performed post-acquisition by exporting the acquired image sets using commercial software or custom scripts (e.g., MATLAB, RRID, SCR_001622)*.
  - Acquire time-lapse recordings at 1-10 Hz depending on the experiment.
    *Generally, activity that produces subtle, fast changes in calcium influx should be recorded at 10 Hz, whereas activities that produce large, robust calcium in fluxes can be recorded at 1 Hz. The length of recording will depend on the application, i.e., drug perfusions, number of treatments, and latency of response, but is typically 30-50 min*.
  - Near the end of each recording, perfuse cultures with recording solution containing 30 mM KCl. This will depolarize the cells and act as a benchmark for the electrical maturity and general health of the cells. An example recording is shown in Figure 4C.

***ALTERNATE PROTOCOL***

## Calcium Imaging of Green Fluorescent Hypothalamic Neurons Using Rhod-3 AM

Rhod-3 AM is a calcium dye with excitation and emission peaks of 560 and 600 nm, respectively. Like other red-shifted calcium dyes, Rhod-3 AM displays low basal autofluorescence and reduced phototoxicity and is compatible with the simultaneous detection of green fluorescent proteins or other green dyes. Below we describe a protocol to monitor calcium responses from hPSC-derived hypothalamic neurons that have been labeled with a green fluorescent reporter by exploiting the multiplexing property of Rhod-3 AM.

*NOTE:* For the fluorescence imaging system, a number of options are available to the user, and the configuration below serves as a guide. Most importantly, the illumination and filters need to be suitable for the intended fluorophores, the objective needs to be of sufficient magnification and numerical aperture (NA) to efficiently collect emitted photons, and the camera should be fast and sensitive enough to detect these photons. Software to control shutters and collect movies that can then be analyzed in R or similar analysis software is also key.

*CAUTION:* If using synaptic blockers, handle each of these toxins with great care and appropriate personal protective equipment and only after carefully familiarizing yourself with the potential risks by reading the Material Safety Data Sheets and discussing their use with your local safety officer.

### Materials

hPSC-derived hypothalamic neurons, Day 30+ (preferably Day 40-60; see Basic Protocol 3), optionally generated from a cell line with a green fluorescent reporter for a cell type of interest
35-mm Ibidi dishes (Thistle Scientific, cat. no. 80136), coated with Geltrex (see Basic Protocol 1m step 1)
BrainPhys™ medium (Bardy et al., 2015; STEMCELL Technologies, cat. no. 05790)
Recording solution: HBSS supplemented with synaptic blockers or BrainPhys™ Imaging optimized neuronal medium (Zabolocki et al., 2020; STEMCELL Technologies, cat. no. 05796; other media, such as ACSF or BPI, can be substituted if desired)
Rhod-3 AM loading buffer (see recipe)
Incubation buffer: HBSS containing 2.5 mM probenecid (Included in the Rhod-3 Calcium Imaging Kit, Thermo Fisher Scientific, cat. no. R10145).
Synaptic blockers (see recipe)

Perfusion system: we use a custom-built gravity perfusion system, but commercial systems are also available, e.g., MPS-2 (World Precision Instruments)
Inverted phase-contrast microscope
37°C, 20% O_2_, 5% CO_2_ humidified incubator
Fluorescence imaging system: e.g.,
  Olympus BX51WI Fixed Stage Upright Microscope (RRID:SCR_023069)
  CoolLED pE-300white (RRID:SCR_021073) illumination system. Emission filters: 508 for GFP (e.g., BrightLine® single-band filter set, GFP-A-Basic-000 (FF01-469/35-25, FF01-525/39-25, and FF497-Di01-25 × 36) and 600 nm (e.g., BrightLine® single-band filter set, mCherry-C-000 (FF01-562/40-25, FF593-Di03-25 × 36, and FF02-641/75-25) for Rhod-3 AM
  Objective: Olympus LUMPLFLN 40XW
  sCMOS camera 16-bit high-speed ORCA Flash4.0 LT plus (RRID:SCR_021971)
Imaging software: HCImage (RRID:SCR_015041)

Additional reagents and equipment for Geltrex coating of plates (Basic Protocol 1, step 1).

1. Prepare cells for calcium imaging.
  - Seed neurons at 1-1.5 × 10^5^ cells per cm^2^ onto Geltrex-coated 35-mm Ibidi dishes (see Basic Protocol 3, step 2) in 1 ml SJ2.
  - Maintain the cells in a tissue culture incubator at 37°C with 5% CO_2_.
  - 24-48 hr before imaging, replace SJ2 with 1 ml warm (37°C) BrainPhys™ and maintain the cells in a tissue culture incubator at 37°C with 5% CO_2_.
2. Perform loading protocol.
  - Gently remove medium and wash twice with warm (37°C) recording medium (e.g., HBSS).
  - Incubate for 30-60 min in the dark with 2 ml of Rhod-3 AM loading buffer at 37°C, and wash cells twice with warm (37°C) HBSS.
  - Add 2 ml warm (37°C) incubation buffer (HBSS containing 2.5 mM probenecid) and incubate cells at room temperature in the dark for 30-60 min at 37°C.
  - Wash cells twice in warm (37°C) HBSS.
  - Add 2 ml of warm (37°C) HBSS. The cells are now ready for calcium imaging experiments.
3. Perform calcium imaging.
  - If perfusion is required, prepare perfusion system during the final incubation after loading the cells with Rhod-3 AM. Set up so that perfusion flows at a rate of ~3 ml/min.
    *To record the intrinsic calcium responses from individual neurons, add synaptic blockers to the HBSS solution (see recipe and Fura-2 AM protocol for details)*.
  - Place imaging dishes on the stage of the imaging microscope (here we used an Olympus BX51WI Fixed Stage Upright Microscope) and wait 10 min for perfusion and temperature to stabilize.
  - Identify a field containing a non-confluent monolayer of cells. Detect the presence of green-positive cells by using an excitation wavelength to 395 nm and emission filter at 508 nm. Switch excitation wavelength to 560 nm and emission filter to 600 nm to determine whereas the GFP-positive cells display a basal autofluorescence for Rhod-3 AM.
  - Use software for acquisition that simultaneously controls the camera and the illumination system. Input an acquisition protocol to excite the cells at 560 nm and acquire at 600 nm at the rate of 1 frame/s (100 ms exposure/frame).

## Reagents and Solutions

*For culture recipes and steps, use sterile tissue-culture-grade water. For other purposes, use deionized, distilled water or equivalent in recipes and protocol steps*.

### ACSF

129 mM NaCl (Sigma-Aldrich, cat. no. 31434)
5 mM KCl (Sigma-Aldrich, cat. no. 31248)
1 mM CaCl_2_ (Sigma-Aldrich, cat. no. C5670)
1 mM MgCl_2_ (Promega, cat. no. A3511)
25 mM HEPES (Thermo Fisher Scientific, cat. no. 15630056)
11.1 mM glucose (Sigma-Aldrich, cat. no. G7021)
Check that pH is 7.2-7.4 and adjust if necessary
Store up to 8 weeks at 4°C
  *Stock solutions of each reagent are made up in sterile distilled water*.

### ACSF with 30 mMKCl

104 mM NaCl (Sigma-Aldrich, cat. no. 31434)
30 mM KCl (Sigma-Aldrich, cat. no. 31248)
1 mM CaCl_2_ (Sigma-Aldrich, cat. no. C5670)
1 mM MgCl_2_ (Promega, cat. no. A3511)
25 mM HEPES (Thermo Fisher Scientific, cat. no. 15630056)
11.1 mM glucose (Sigma-Aldrich, cat. no. G7021)
Check that pH is 7.2-7.4 and adjust if necessary
Store up to 8 weeks at 4°C
  *Stock solutions of each reagent are made up in sterile distilled water*.

### Brain-derived neurotrophic factor (BDNF), 100 μg/ml (10,000×) stock

Reconstitute BDNF powder (PeproTech, cat. no. 450-02) in DPBS^–^ (Thermo Fisher Scientific, cat. no. 14190250) with 0.1% BSA (Sigma-Aldrich, cat. no. A0281; sterile filtered) to 100 μg/ml, divide into aliquots, and store at –80°C. Use at 1:10,000 for a 10 ng/ml final concentration.

### DAPI, 3.6 mM (10,000×) stock

Reconstitute DAPI powder (Sigma-Aldrich, cat. no. 10236276001) in sterile distilled water to 3.6 mM (1 mg/ml) in the dark at room temperature and store in aliquots at –20°C. Use at 1:10,000 for a 360 nM final concentration.

### DAPT, 50 mM (10,000×) stock

Reconstitute DAPT powder (Sigma-Aldrich, cat. no. D5942) in DMSO (Sigma-Aldrich, cat. no. D2650-100 ml) to generate a 50 mM stock, aliquot, and store in aliquots at –20°C. Use at 1:10,000 for a 5 μM final concentration.

### FACS sorting buffer

800 μl of 7.5% bovine serum albumin (Sigma-Aldrich, cat. no. A0281; sterile filtered)
60 μl of 0.5 M EDTA (Invitrogen, cat. no. 15575020)
750 μl of 1 M HEPES (Sigma-Aldrich, cat. no. H0887)
DPBS^–^ (Thermo Fisher Scientific, cat. no. 14190250), pH 7.0, to 30 ml

### FACS wash buffer

15 ml basal medium (e.g., Synaptojuice 2)
15 μl Y-27632 dihydrochloride ROCK inhibitor stock solution (1000×; see recipe; final concentration, 10 μM)
15 μl of 30 mM actinomycin D (Sigma, cat. no. A1410)
1 vial D2 DNase (Worthington, cat. no. LK003170)

### FGF2, 20 μg/ml (1000×) stock

Reconstitute FGF2 powder (Sigma-Aldrich, cat. no. F0291) according to manufacturer’s directions to generate a 20 μg/ml stock, divide into aliquots, and store at – 80°C. Use at 1:1000 for at 20 ng/ml final concentration.

### Freezing medium (2×)

Prepare 2×freezing medium by slowly adding 50 ml of 100% DMSO (tissue-culture grade) to 200 ml of ice-cold fetal bovine serum. Mix well in a plastic or glass container, sterile filter with a 0.22-μm-pore-size filter flask, and store up to 6 months at 4°C. Avoid adding DMSO directly onto the filter.

### Fura-2AM, 1 mM (3000×) stock

Prepare Fura-2AM stock by dissolving 50 μg Fura-2AM (Thermo Fisher, cat. no. F1221) in 48 μl DMSO (Sigma-Aldrich, cat. no. D2650-100 ml) with 2 μl of 10% w/v pluronic acid (Sigma-Aldrich, cat. no. P2443). This will yield a 1 mM stock solution. Aliquot and freeze at –20°C.

### Fura-2AM loading solution

Add 1 μl of 1 mM Fura-2AM stock solution (see recipe) per 3 ml ACSF (333 nM final concentration). Prepare 1 ml per 35-mm dish to be loaded. Use fresh—do not store long term.

### hPSC wash medium

30 ml StemFlex medium (see recipe)
30 μl Y-27632 dihydrochloride ROCK inhibitor stock solution (1000×; see recipe; final concentration, 10 μM)
1 vial D2 DNase (Worthington, cat. no. LK003170)
Use fresh—do not store long term

### LDN-193189, 1 mM (10,000×) stock

Reconstitute LDN-193189 powder (Stemgent, cat. no. 04-0074) in DMSO (Sigma- Aldrich, cat. no. D2650-100 ml) according to manufacturer’s directions to generate a 1 mM stock, aliquot, and store aliquots at −20°C. Use at 1:10,000 for a 100 nM final concentration.

### Maturation medium

1 L N2B27 medium (see recipe)
BDNF (see recipe for 100 μg/ml stock) to 10 ng/ml final concentration, added fresh at each feeding
Store at 4°C; do not store with BDNF for >48 hr

### N2B27 medium

500 ml Neurobasal-A (Thermo Fisher Scientific, cat. no. 10888022)
500 ml DMEM/F12 with GlutaMAX (Thermo Fisher Scientific, cat. no. 31331093)
10 ml GlutaMAX (Thermo Fisher Scientific, cat. no. 35050038)
10 ml sodium bicarbonate (Thermo Fisher Scientific, cat. no. 25080-094)
5 ml MEM Nonessential Amino Acids (Thermo Fisher Scientific, cat. no. 11140035)
1 ml of 200 mM ascorbic acid (use at 1:1000; Sigma-Aldrich, cat. no. A4403)
10 ml of 100 × penicillin-streptomycin (Thermo Fisher Scientific, cat. no. 15140122)
Sterile filter, then add the following supplements:
20 ml of 50× B27 supplement (Thermo Fisher Scientific, cat. no. 17504044)
10 ml of 100× N2 supplement (Thermo Fisher Scientific, cat. no. 17502048)
Store up to 1 week at 4°C

### Rhod-3 AM loading buffer

20 μl of 100× PowerLoad™ concentrate
2 μl of 10 mM Rhod-3 AM (Rhod-3 Calcium Imaging Kit, cat. no. R10145; Thermo Fisher Scientific)
Vortex to mix
HBSS to 2 ml
20 μl of 250 mM Probenecid

### Purmorphamine, 10 mM (10,000×) stock

Reconstitute purmorphamine powder (Calbiochem, cat. no. 540220) in DMSO (Sigma-Aldrich, cat. no. D2650-100 ml) to generate a 10 mM stock, divide into aliquots, and store at –80°C. Use at 1:10,000 for a 1 μM final concentration.

### SB431542, 10 mM (1000×) stock

Reconstitute SB431542 powder (Sigma-Aldrich, cat. no. S4317) in DMSO to generate a 10 mM stock, divide into aliquots, and store aliquots at –80°C. Use at 1:1000 for a 10 μM final concentration.

### Smoothened agonist (SAG), 10 mM (10,000×) stock

Reconstitute SAG powder (Fisher Scientific, cat. no. 56-666-01MG) in DMSO (Sigma-Aldrich, cat. no. D2650-100 ml) to generate a 10 mM stock, divide into aliquots, and store at –80°C. Use at 1:10,000 for a 1 μM final concentration.

### StemFlex medium

450 ml StemFlex basal medium (Thermo Fisher Scientific, cat. no. A3349401)
50 ml StemFlex supplement (defrost before use)
Store at 4°C; protect from light

### Synaptic blocker stock solutions

*CAUTION:* Handle each of these toxins with great care and appropriate personal protective equipment and only after carefully familiarizing yourself with the potential risks by reading the Material Safety Data Sheets and discussing their use with your local safety officer.

Divide the following stocks into aliquots and store at −20°C.

1. Dissolve picrotoxin (cat. no. 1128; Tocris) to a stock concentration of 10 mM in DMSO. Use at 1:200 for a final concentration of 50 μM.
2. Dissolve strychnine hydrochloride (cat. no. ab120416; Abcam) to a stock concentration of 50 mM in sterile water. Use at 1:2500 for a final concentration of 20 μM
3. Dissolve DL-AP5 sodium salt (cat. no. ab120271; Abcam) to a stock concentration of 50 mM in sterile water. Use at 1:500 for a final concentration of 100 μM.
4. Dissolve CNQX disodium salt (cat. no. ab120044; Abcam) to a stock concentration of 30 mM in sterile water. Use at 1:1000 for a final concentration of 30 μM.

### Synaptojuice 1 (SJ1) enhanced maturation medium

500 ml N2B27 medium (see recipe)
50 μl of 50 mM DAPT (Sigma-Aldrich, cat. no. D5942)
50 μl of 20 mM PD0332991 Isethionate (Sigma-Aldrich, cat. no. PZ0199)
50 μl of 20 mM CHIR99021 (Cell Guidance Systems, cat. no. SM13-10)
50 μl of 10 mM LM22A4 (Tocris, cat. no. 4607)
185 μl of 1 M calcium chloride (Sigma-Aldrich, cat. no. 21115)
500 μl of 10 mM NHK477 (Sigma-Aldrich, cat. no. N3290)
500 μl of 300 mM GABA (Tocris, cat. no. 0344)
BDNF (see recipe for 100 μg/ml stock) to 10 ng/ml final concentration, added fresh at each feeding
Store at 4°C; do not store with BDNF for >48 hr

### Synaptojuice 2 (SJ2) enhanced maturation medium

500 ml N2B27 medium (see recipe)
50 μl of 20 mM PD0332991 Isethionate (Sigma-Aldrich, cat. no. PZ0199)
50 μl of 20 mM CHIR99021 (Cell Guidance Systems, cat. no. SM13-10)
50 μl of 10 mM LM22A4 (Tocris, cat. no. 4607)
185 μl of 1 M calcium chloride (Sigma-Aldrich, cat. no. 21115)
500 μl of 300 mM GABA (Tocris, cat. no. 0344)
BDNF (see recipe for 100 μg/ml stock) to 10 ng/ml final concentration, added fresh at each feeding
Store at 4°C; do not store with BDNF for >48 hr

### Tris-buffered saline (TBS)

Dissolve TBS tablets (Sigma-Aldrich, cat. no. T5030) in sterile distilled water according to manufacturer’s directions. Adjust the pH to 7.4 with hydrochloric acid. Store at room temperature.

### Trituration medium

30 ml maturation medium (see recipe)
30 μl Y-27632 dihydrochloride ROCK inhibitor stock solution (1000×; see recipe) to 10 μM final concentration
1 vial D2 DNase (Worthington, cat. no. LK003170)
Use fresh—do not store long term

### XAV939, 10 mM (5000×) stock

Reconstitute XAV939 powder (Stemgent, cat. no. 04-0046) in DMSO (Sigma-Aldrich, cat. no. D2650-100 ml) according to manufacturer’s directions to generate a 10 mM stock, divide into aliquots, and store aliquots at –20°C. Use at 1:5000 for a 2 μM final concentration.

### Y-27632 dihydrochloride (ROCK inhibitor), 10 mM (1000×) stock

Reconstitute Y-27632 dihydrochloride powder (DNSK International, cat. no. DNSK-KI-15-02) in sterile distilled water to generate a 10 mM stock, divide into aliquots, and store aliquots at –20°C. Use at 1:1000 for a 10 μM final concentration.

## Commentary

### Background Information

The aim of this unit is to enable research groups with adequate facilities and expertise to generate hPSC-derived hypothalamic neurons. Complemented with *in vivo* validation in animal models, the utility of this hPSC system should prove a powerful tool to study the basic biology of hypothalamic neurons and to model diseases of hypothalamic origin with the aim of developing improved treatments.

A principal advantage of this culture system is its scalability. Because each 10-cm plate of stem cells yields ~1 × 10^8^ neurons or more, even neuron types that are produced relatively inefficiently (~1%) can be obtained in large numbers (~1 × 10^6^ per plate). This enables human hypothalamic neurons to be deeply characterized, used in disease modeling studies, and studied in high-throughput imaging assays and analyses, paving the way for small-molecule screens for modulators of hypothalamic neuron activity and function.

Furthermore, depending on the application, experiments can be carried out at relatively early time points such as Day 30 to Day 40 (Pantazis et al., 2022). This allows the study of certain cell-intrinsic responses, in contrast to other culture system in which electrophysiological maturation can take several months (Hartfield et al., 2014; Kirwan et al., 2015; Shi et al., 2012).

Another key advantage of the hPSC system is the ability to perform gene editing using CRISPR/Cas9 (Cong et al., 2013; Santos et al., 2016). Gene-editing technology should provide a powerful platform to probe key aspects of human hypothalamic biology and investigate the mechanisms of metabolic disorders. Together, these techniques could allow a robust investigation of the molecular and cellular pathways that are important for hypothalamic health and disease.

### Critical Parameters

#### hPSC culture

It is critical that hPSCs be passaged routinely and not be allowed to become over-confluent or contaminated with differentiated cells. Because hPSCs accumulate genetic defects over time in culture (Amps et al., 2011; Merkle et al., 2017; Ronen & Benvenisty, 2012), it is advisable to use cell lines at low to moderate passage number (<P50). Avoid splitting cells at ratios exceeding 1:10, as sharp reductions in the size of a cell population can lead to unwanted selection events. We also recommend working with cell lines that have been rigorously genetically characterized by sequencing, and routinely karyotyping and/or sequencing cell lines to test for the acquisition of unwanted mutations.

#### Cell density and feeding frequency

Major causes of cell death or poor differentiation can be cell overgrowth or inadequate nutrient availability. First, ensure that initial plating densities are accurate by using automated cell counters that adjust for the number of live cells. There is some variability in growth rate between cell lines. The protocols described here should be appropriate for most cell lines, but if you notice significant cell death or find that your medium visibly acidifies within 24 hr when phenol red is present as an indicator, feed cells more frequently or with larger medium volumes.

#### Cell passaging

Cell passaging can be a major source of cell death. It is essential to handle cells gently when mechanically triturating them, particularly stem cells and neurons. Minimize the formation of bubbles by aspirating and expelling less than the full medium volume. Do not use excessive force when pipetting, and ensure that cells easily wash off of the plate before attempting to dissociate the culture, increasing the time that cells are incubated in EDTA, TrypLE, or TrypLE + papain, if necessary. Ensure that DNase is present in the trituration medium.

### Troubleshooting

#### Poor neural specification

At Day 10 to Day 14, the majority of cells (>80%) should stain positive for the ventral forebrain progenitor transcription factor NKX2.1. Between Days 15 and 30, neurons should become the dominant cell type in the cultures. If this does not occur, there was probably a defect in neuronal patterning and specification. The likely causes are:

1. *Poor-quality or differentiated hPSCs:* Make sure that hPSCs are maintained appropriately, lack differentiated cells, and are in rapid growth phase when they are dissociated and plated for differentiation (refer to Critical Parameters and Basic Protocol 1)
2. *Low starting density:* Make sure the density of hPSCs is ≥60%.
3. *Reagent problem:* Make sure the differentiation medium is fresh. Avoid using N2B27 medium older than 2 weeks. Add small-molecule inhibitors and recombinant proteins on the day of use. Avoid repeated freeze-thaw cycles of aliquots and other reagents.

#### Contaminating cell types during differentiation

Some non-neuronal cell types can undergo significant proliferation and become dominant in older cultures. If this is the case, based on immunostaining and phase-contrast microscopy, extend DAPT treatment to Day 20.

#### Cell detachment

Detachment can sometimes occur during the early phase of hypothalamic differentiation. Ensure that cells are ~75% confluent when Day 0 medium is added. Poor Geltrex coating can also result in detachment. Make sure Geltrex is always thawed at 4°C and generate single-use aliquots. Do not use Geltrex that contains visible clumps, and use plates immediately after coating. Laminin (final concentration: 1 μg/ml) may be supplemented in during the maturation stage to provide better cell attachment. Detachment can also occur in older cultures (>Day 50). Make sure that the density at the terminal plating is not <5 × 10^4^ cells per cm^2^ or >2 × 10^5^ cells per cm^2^. Dissociate and re-plate cells, if necessary, with Try- pLE + papain.

#### Neurons do not attach after passaging

Make sure TrypLE is fully washed out, as residual enzyme can prevent attachment. Ensure that plates are properly coated with Geltrex (see above). If plating on glass, make sure the glass is thoroughly acid washed. Poor neuronal survival can also indicate overdigestion in TrypLE or TrypLE + papain, or rough mechanical dissociation (see Critical Parameters).

Cultures older than Day 40 should be replated at lower density to aid neuronal survival, as other non-neuronal cell types can undergo significant proliferation. Neurons can also be purified by FACS if a reporter line is available or using a neuron-specific antigen such a NCAM.

#### Cell death in older cultures

When cell density increases, particularly as the cultures become older, the culture medium can acidify and become depleted of nutrients. Feed cultures daily or with a larger medium volume, or re-plate if necessary.

### Statistical Analysis

To allow thorough statistical analysis, all experiments should be performed with a minimum of three replicate wells. In addition, experiments should be repeated in independent differentiations, preferably with three genetically distinct cell lines. The statistical analysis performed will vary depending on the experimental design.

### Understanding Results

If the procedures for quality control are followed, it should be straightforward for users to determine whether they have succeeded in generating hypothalamic neurons, which share a number of key features with their *in vivo* counterparts (Merkle et al., 2015; Wang et al., 2015, 2017).

### Time Considerations

Expanding hPSCs for differentiation typically takes 1-2 weeks. Differentiation to NKX2.1-positive progenitors takes 8-10 days. Hypothalamic neurons are generated after 14-20 days. POMC immunopositive cells are clearly visibly by Day 25. Functional studies such as calcium imaging can be carried out as soon as Day 30, although further culturing cells to Day 60 may increase neuronal maturation and functional responsiveness. With careful maintenance, cultures can be maintained for well over 100 days.

## Figures and Tables

**Table 1:** Stock and Working Concentrations of Reagents Used to Differentiate Hypothalamic Neurons

Hypothalamic neuron differentiation protocol (in 50 ml of N2B27)													
Molecule	Stock conc.	Use at	Day 0			Day 2			Day 4			Day 6	
			Work conc.	Vol. (μl)		Work conc.	Vol. (μl)		Work conc.	Vol. (μl)		Work conc.	Vol (μl)
**XAV**	10 mM	1:5000	2μM	**10**		2μM	**10**		1.5 μM	**7.5**		1 μM	**5**
**LDN**	1 mM	1:10,000	100 nM	**5**		100 nM	**5**		75 nM	**3.75**		50 nM	**2.5**
**SB431542**	10 mM	1:1000	10 μM	**50**		10 μM	**50**		7.5 μM	**37.5**		5 μM	**25**
**SAG**	10 mM	1:10,000				1 μM	**5**		1μM	**5**		1μM	**5**
**Purmor**	10 mM	1:10,000				1 μM	**5**		1 μM	**5**		1 μM	**5**
**DAPT**	50 mM	1:10,000											
			Day 8			Day 10			Day 12			Day 14	
Molecule	Stock conc.	Use at	Work conc.	Vol. (μl)		Work conc.	Vol. (μl)		Work conc.	Vol. (μl)		Work conc.	Vol (μl)
**XAV**	10 mM	1:5000	0.5 μM	**2.5**									
**LDN**	1 mM	1:10,000	25 nM	**1.25**									
**SB431542**	10 mM	1:1000	2.5 μM	**12.5**									
**SAG**	10 mM	1:10,000											
**Purmor**	10 mM	1:10,000											
**DAPT**	50 mM	1:10,000	5 μM	**5**		5μM	**5**		5μM	**5**		5μM	**5**

**Table 2:** Stock and Working Concentrations of Reagents Used to Differentiate Cortical Neurons

Cortical neuron differentiation protocol (in 50 ml N2B27)													
Molecule	Stock conc.	Use at	Day 0			Day 2			Day 4			Day 6	
			Work conc.	Vol. (μl)		Work conc.	Vol. (μl)		Work conc.	Vol. (μl)		Work conc.	Vol. (μl)
**XAV**	10 mM	1:5000	2 μM	**10**		2 μM	**10**		1.5 μM	**7.5**		1 μM	**5**
**LDN**	1 mM	1:10,000	100 nM	**5**		100 nM	**5**		75 nM	**3.75**		50 nM	**2.5**
**SB431542**	10 mM	1:1000	10 μM	**50**		10 μM	**50**		7.5 μM	**37.5**		5 μM	**25**
**FGF2**	20 μg/Ml	1:1000											
			Day 8			Day 10			Day 12			Day 14	
Molecule	Stock conc.	Use at	Work conc.	Vol. (μl)		Work conc.	Vol. (μl)		Work conc.	Vol. (μl)		Work conc.	Vol. (μl)
**XAV**	10 mM	1:5000	0.5 μM	**2.5**									
**LDN**	1 mM	1:10,000	25 nM	**1.25**									
**SB431542**	10 mM	1:1000	2.5 μM	**12.5**									
**FGF2**	20 μg/Ml	1:1000							20 ng/ml	**50**		20 ng/ml	**50**

**Table 3:** Proteins Detectable by Immunofluorescence in Differentiated Cultures by Day 14 or by Day 40

Expression	Hypothalamic neurons	Cortical neurons
Day 14	FOXG1 (few cells) NKX2.1 (most cells)	FOXG1 (most cells) NKX2.1 (few cells) OTX1/2 (many cells) PAX6 (many cells)
Day 40	TUJ1 (most cells) MAP2 (most cells) POMC (many cells) AGRP (some cells) HCRT (some cells) PMCH (some cells)	TUJ1 (most cells) MAP2 (most cells) POMC (negative) AGRP (negative) TBR1 (many cells) CTIP2 (many cells)

**Table 4:** List of Primary Antibodies

Antigen	Supplier, cat. no., RRID	Species	Dilution
AGRP	Phoenix Pharmaceuticals, cat. no. H-003-53, lot no. RRID:AB_2313908	Rabbit	1:500
CTIP2	Abcam, cat. no. ab18465, lot no. RRID:AB_2064130	Rat	1:500
FOXG1	Abcam, cat. no. ab196868, lot no. RRID:AB_2892604	Rabbit	1:100
MAP2	Abcam, cat. no. ab5392, lot no. RRID:AB_2138153	Chicken	1:2000
NKX2.1	Thermo Fisher Scientific, cat. no. 18-0221, lot. no RRID:AB_86728	Mouse	1:500
OTX1/2	Millipore, cat. no. AB9566, lot no. RRID:AB_2157186	Rabbit	1:300
PAX6	BioLegend, cat. no. 901301, lot no. RRID: AB_2565003	Rabbit	1:500
POMC	Thermo Fisher Scientific, cat. no. MA5-38604, lot no. RRID: AB_2898516	Mouse	1:200
PMCH	Sigma-Aldrich, cat. no. M8440, lot no. RRID:AB_260690	Rabbit	1:1000
TBR1	Abcam, cat. no. ab31940, lot no. RRID:AB_2200219	Rabbit	1:500
TUJ1	BioLegend, cat. no. 801201, lot no. RRID: AB_2313773	Mouse	1:1000

**Table 5:** Primers Used for Gene Expression Analysis

Gene	Expected expression in hypothalamic cultures	Expected expression in cortical cultures	TaqMan assay ID (Thermo Fisher)
*AGRP*	High	Low	Hs00361403_g1
*EMX1*	Low	High	Hs00417957_m1
*FOXG1*	Low	High	Hs01850784_s1
*NKX2.1*	High	Low	Hs00968940_m1
*POMC*	High	Low	Hs01596743_m1
*OTP1*	High	Low	Hs01888165_s1
*SIM1*	High	Low	Hs00231914_m1

## Data Availability

Data sharing not applicable—no new data generated.
